# Predicting Dengue Outbreaks in Cambodia

**DOI:** 10.3201/eid2512.181193

**Published:** 2019-12

**Authors:** Anthony Cousien, Julia Ledien, Kimsan Souv, Rithea Leang, Rekol Huy, Didier Fontenille, Sowath Ly, Veasna Duong, Philippe Dussart, Patrice Piola, Simon Cauchemez, Arnaud Tarantola

**Affiliations:** Institut Pasteur du Cambodge, Phnom Penh, Cambodia (A. Cousien, J. Ledien, K. Souv, D. Fontenille, S. Ly, V. Duong, P. Dussart, P. Piola);; Institut Pasteur, Paris, France (A. Cousien, S. Cauchemez);; CNRS, Paris (A. Cousien, S. Cauchemez);; National Center for Entomology, Parasitology and Malaria Control, Phnom Penh (R. Leang, R. Huy);; Institut Pasteur, Noumea, New Caledonia (A. Tarantola)

**Keywords:** Pediatric dengue, dengue, modeling, Cambodia, surveillance data, magnitude of the epidemic, healthcare system, viruses

## Abstract

In Cambodia, dengue outbreaks occur each rainy season (May–October) but vary in magnitude. Using national surveillance data, we designed a tool that can predict 90% of the variance in peak magnitude by April, when typically <10% of dengue cases have been reported. This prediction may help hospitals anticipate excess patients.

Dengue is endemic to Cambodia; outbreaks are seasonal, occurring during the rainy season (May–October). However, the magnitude of outbreaks varies from year to year. When the epidemic is particularly large, the influx of patients with severe dengue in pediatric hospitals may saturate the healthcare system and negatively affect quality of care. However, adequate supportive care is crucial for patients with severe dengue and can decrease the fatality rate to <1% (*1*). Early prediction of the size of nascent dengue epidemics may improve healthcare planning and optimize allocation of healthcare resources. We used surveillance data to build a simple early warning tool based on the reported number of cases early in the season. Compared with other approaches used to predict dengue epidemics (*2**–**6*), this one is characterized by its simplicity because it relies only on the number of cases reported early in the season to predict the magnitude of the epidemic.

## The Study

We used the monthly number of probable dengue cases reported by the National Dengue Surveillance System (NDSS) in Cambodia during 2004–2016. The NDSS includes passive surveillance of probable dengue pediatric inpatients reported by public hospitals to the Communicable Diseases Center of the Ministry of Health and a sentinel, pediatric hospital–based active surveillance system managed by the National Dengue Control Program of the National Center for Parasitology, Entomology and Malaria Control, Ministry of Health. A probable dengue case was defined as an acute febrile illness with >2 of the following: headache, retro-orbital pain, myalgia, arthralgia, rash, hemorrhage, and leukopenia, combined with either 1) a posteriori virologic confirmation, serologic confirmation, or both or 2) presence of >1 laboratory-confirmed case at the same location and time (*7*).

**Figure 1:**
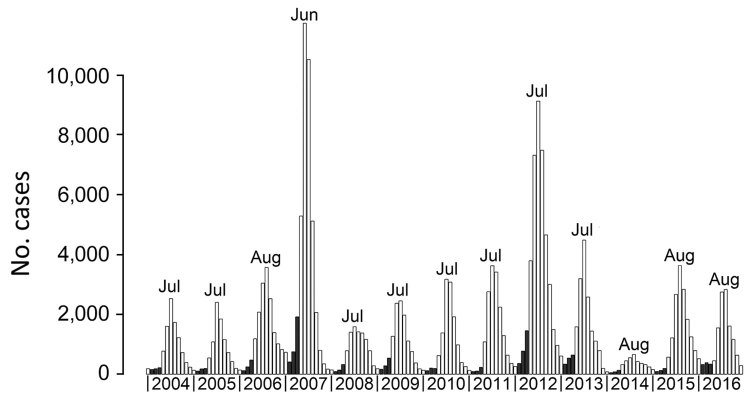
Monthly number of probable dengue cases reported to the National Dengue Surveillance System in Cambodia, 2004–2016. Dark gray bars represent the 3 months (February, March, and April) used as predictors for the magnitude of the following peak. For each year, the month corresponding to the peak of the epidemic is indicated.

From January 2004 through December 2016, NDSS reported 215,574 probable dengue cases (Figure 1). During this period, we observed 2 outbreaks of particularly high magnitude, in 2007 (dengue virus serotype 3) and 2012 (dengue virus serotype 1). The magnitude of these outbreaks reached ≈10,000 cases versus the usual number of <5,000 cases. Incidence was always lowest during the dry season (i.e., November–April); the nadirs usually occurred in February and the peaks in July (8 times), August (4 times), and June (1 time, in 2007). On average, only 6.1% of the cases reported during a season (i.e., from February through January of the following year) are observed before the end of April (range 2.7%–9.0% of cases). We wanted to ascertain whether the small number of cases reported at the season’s onset (i.e., up to April) could be used as an early warning tool for predicting the magnitude of that season’s epidemic.

**Figure 2:**
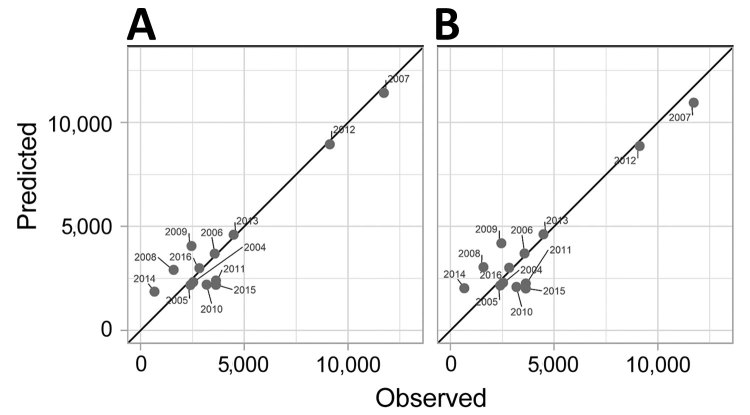
Dengue cases in Cambodia, 2004–2016. A) Observed versus predicted magnitude of the peak for each dengue season. We used a simple linear regression model, *M* = α + β*N*, in which *M* indicates the magnitude of the peak and *N* the number of reported dengue-like cases in April. The black line represents the expected results with perfect prediction. B) Results for the leave-one-out cross-validation procedure.

We observed a strong linear correlation between the magnitude of the peak and the number of cases reported at the beginning of the season, in February (Pearson correlation coefficient r = 0.78), March (r = 0.88), April (r = 0.95), February–March (r = 0.86), March–April (r = 0.95), and February–April (r = 0.94). Fitting a simple linear regression model to the data, we estimated that the number of cases reported explained the following parts of the variance in the peak magnitude for February (61%), March (78%), April (91%), February–March (73%), March–April (90%), and February–April (88%). The magnitude was therefore best predicted by the number of dengue cases reported in April. This simple model offered excellent accuracy for predicting the magnitude of the peak; mean absolute percentage error for 2007 was 2.5% and for 2012 was 1.9% (Figure 2, panel A). Predictions relying on data from March were also acceptably accurate; the error was larger, but the model was able to predict a larger than usual magnitude (Appendix Figure 1).

To evaluate the performance of our model in a real-life situation, when the outcome of the ongoing epidemic remains unknown, we used a leave-one-out cross-validation procedure (*8*–*10*). We obtained the predicted value for season *s* by fitting our regression model to the 12 other seasons (i.e., excluding season *s* from the set of observations used to fit the parameters of the model [the training dataset]). The predictive power of our best fitting model remained very high; it was able to explain 90% of the variance of the magnitude of epidemics (Figure 2, panel B).

Our dataset contains information for only 2 large epidemics (2007 and 2012). If we trained the model on these 2 large epidemics only, performance would remain very good (98% variance explained). In contrast, when both epidemics were excluded from the training dataset, their magnitude was underestimated by 35% (2007) and 32% (2012). As expected, to be properly calibrated, the model needs to be trained on a mix of small and large epidemics; if 1 category is excluded from the training dataset, performance may be substantially degraded. 

Of note, this loss of accuracy is mostly an issue for large epidemics. Given the small number of such epidemics in our dataset, robustly demonstrating predictability from this dataset alone remains difficult. We therefore explored whether similar patterns could be observed in 4 other countries in South Asia: Thailand, Vietnam, Laos, and the Philippines (*11*–*14*). To be comparable with our analysis for Cambodia, we used the month at which >5% of cases have been observed on average (Appendix Tables 4, 5). The results were promising for Vietnam (variance explained in the leave-one-out procedure was 64.3%), the Philippines (45.8%), and Thailand (33.4%) but bad for Laos (–53.5%) (Appendix Figures 3–6). This variability could be explained by several factors: national surveillance system characteristics, demographics, land cover, healthcare systems, or climate; all of these factors can affect dengue epidemiology and reporting. This analysis confirms the observation made for Cambodia that the number of dengue cases reported early in the epidemic year may provide early insight into the probable scale of the forthcoming epidemic.

## Conclusions

The correlation between the number of patients hospitalized with probable dengue during the interepidemic period (i.e., the dry season) and the magnitude of the next outbreak peak during the rainy season was strong, even from February, which corresponds to the nadir of the incidence curves. Using dengue surveillance data for the end of the dry season (April), we were able to predict the magnitude of the peak for the next dengue outbreak, when typically <10% of cases have been observed and the peak is 2–3 months away. These results suggest that the intensity of rainfalls during the rainy season is not a major determinant of the occurrence of major outbreaks in Cambodia and that the outbreaks could be explained by conditions already present during the early stages of the outbreak (i.e., the part of the population immune to the circulating strains or weather conditions during the dry season). Our analysis is limited by the small number of epidemic seasons that are available to train our model for Cambodia (in particular, the small number of large epidemics), but similar patterns were observed in some other countries in South Asia.

In a setting where resources are limited and where pediatric hospitals face several other health issues (diarrheal diseases, other infectious diseases), the amount of available beds, medical supplies, and medical staff are usually appropriate for an average dengue outbreak. This simple and easy tool can help hospitals to plan in accordance with the predicted magnitude of the seasonal outbreak.

Appendix Supplementary results for study of predicting dengue outbreaks in Cambodia.
