# Supplementary material for: Predicting Dengue Outbreaks in Cambodia
Source: Emerg Infect Dis. 2019 Dec;25(12):2281–3. doi: 10.3201/eid2512.181193 (PMC6874239; doi:10.3201/eid2512.181193)
Supplement: Appendix — Supplementary results for study of predicting dengue outbreaks in Cambodia. [file 18-1193-Techapp-s1.pdf]

Article DOI: https://doi.org/10.3201/eid2512.181193

Predicting Dengue Outbreaks in Cambodia

Appendix

Appendix Table 1. Magnitudes, timing of the peak and number of probable dengue cases reported in February, March, April, February-March, March-April and February to April to the National Dengue Surveillance System in Cambodia for seasonal dengue outbreaks between 2004 and 2016.

Year	Magnitude	Timing of the peak	Reported cases in February	Reported cases in March	Reported cases in April	Reported cases in February-March	Reported cases in March-April	Reported cases in February-April
2004	2,530	July	162	191	220	353	411	573
2005	2,403	July	113	180	195	293	375	488
2006	3,566	August	122	254	475	376	729	851
2007	11,726	June	416	756	1,917	1,172	2,673	3,089
2008	1,594	July	89	145	330	234	475	564
2009	2,451	July	172	284	544	456	828	1,000
2010	3,176	July	123	209	198	332	407	530
2011	3,628	July	90	112	234	202	346	436
2012	9,125	July	362	773	1,455	1,135	2,228	2,590
2013	4,483	July	340	545	646	885	1,191	1,531
2014	667	August	54	85	134	139	219	273
2015	3,631	August	97	133	196	230	329	426
2016	2,835	August	333	389	346	722	735	1,068

Appendix Table 2. Predicted magnitudes using the linear model $M = \alpha + \beta N$, with M the magnitude of the peak and N the number of reported probable dengue cases in either February, March, April, February-March, March-April and February to April. The last six columns present the results for the "leave-one-out" procedure, i.e., when the current season of the learning sample is excluded.

	Predicted magnitudes using reported cases						Predicted magnitudes in leave-one-out procedure using reported cases					
Year	in February	in March	in April	in February-March	in March-April	in February-April	in February	in March	in April	in February-March	in March-April	in February-April
2004	3,446	2,599	2,322	2,886	2,353	2,478	3,527	2,607	2,297	2,922	2,333	2,473
2005	2,509	2,473	2,187	2,444	2,217	2,199	2,522	2,481	2,161	2,449	2,194	2,174
2006	2,681	3,321	3,691	3,056	3,558	3,391	2,581	3,299	3,701	3,007	3,557	3,376
2007	8,303	9,074	11,432	8,920	10,921	10,737	6,465	7,503	10,952	7,264	9,926	9,597
2008	2,050	2,072	2,912	2,010	2,596	2,449	2,119	2,136	3,040	2,069	2,702	2,544
2009	3,637	3,665	4,061	3,645	3,933	3,880	3,738	3,768	4,195	3,747	4,056	3,999
2010	2,700	2,805	2,203	2,732	2,338	2,337	2,647	2,768	2,086	2,685	2,241	2,239
2011	2,069	1,694	2,397	1,774	2,107	2,029	1,835	1,387	2,257	1,482	1,915	1,820
2012	7,271	9,269	8,951	8,647	9,236	9,099	6,702	9,362	8,870	8,402	9,295	9,086
2013	6,850	6,656	4,608	6,806	5,308	5,623	7,430	7,064	4,620	7,291	5,393	5,755
2014	1,381	1,384	1,860	1,310	1,626	1,493	1,533	1,515	2,024	1,435	1,772	1,627
2015	2,203	1,935	2,193	1,980	2,043	1,996	2,002	1,692	2,018	1,743	1,838	1,779
2016	6,716	4,868	2,998	5,605	3,580	4,103	7,602	5,059	3,013	5,942	3,644	4,209

Appendix Table 3. Estimated parameters (and 95% confidence intervals) for the linear model $M = \alpha + \beta N$, with M the magnitude of the peak and N the number of probable dengue cases reported to NDSS in either February, March or April, and the corresponding proportion of the variance of M explained.

	Estimates for the model $M = \alpha + \beta N$, with M the magnitude and N the number of cases					
Description	February	March	April	February-March	March-April	February-April
α	348.0 [-1,948.8; 2,644.8]	410.4 [-1,153.7; 1,974.5]	1140.56 [288.8; 1992.3]	285.9 [-1,501.3; 2,073.0]	796.4 [-156.7; 1,749.6]	597.3 [-466.7; 1,661.2]
β	19.1 [8.9; 29.3]	11.5 [7.4; 15.5]	5.39 [4.2; 6.5]	7.4 [4.4; 10.3]	3.8 [2.9; 4.6]	3.3 [2.5; 4.1]
Variance explained by the model*	61%	78%	91%	73%	90%	88%
Variance explained, leave-one-out procedure†	42%	69%	90%	63%	87%	85%

*Corresponding to the squared correlation coefficient r^2.
†Ccorresponding to the predictive squared correlation coefficient q_{CV}^2 obtained in the “leave-one-out” cross validation procedure.

Appendix Table 4. Month with >5% of the total number of cases observed for 5 countries in Southeast Asia. Monthly data for the number of case reported in each dengue national surveillance system (excluding Cambodia) are publicly available in (*1*–*4*).

Country	Month with >5% of the total number of cases observed (%)
Cambodia	April (6.1%)
Thailand	February (7.3%)
Vietnam	March (6.0%)
Laos	March (5.6%)
Philippines	May (8.9%)

Appendix Table 5. Estimated parameters (and 95% confidence intervals) for the linear model $M = \alpha + \beta N$, with M the magnitude of the peak and N the number of probable dengue cases reported in national dengue surveillance systems (*1*–*4*), when 5% of the total number of cases have been observed, and the corresponding proportion of the variance of M explained.

	Estimates for the model $M = \alpha + \beta N$, with M the magnitude and N the number of cases				
Description	Cambodia	Thailand	Vietnam	Laos	Philippines
α	1140.6 [288.8; 1992.3]	5590.1 [1963.2; 9217.1]	–4875.4 [-10889.1; 1138.4]	3228.3 [-250; 6706.6]	–4327.1 [-10142.8; 1488.5]
β	5.4 [4.2; 6.5]	2 [0.8; 3.2]	8.1 [5.6; 10.7]	–3.1 [-28.5; 22.3]	10.2 [6.2; 14.2]
Variance explained by the model*	90.6	42.8	76.2	0.6	74.2
Variance explained, leave-one-out procedure†	89.8	33.4	64.3	–53.5	45.8

*Corresponding to the squared correlation coefficient r^2.
†Corresponding to the predictive squared correlation coefficient q_{CV}^2.

References

1. Van Panhuis W, Cross A, Burke D, Choisy M. Counts of Dengue reported in Philippines: 1955–2010: Project Tycho dataset. University of Pittsburgh; 2018 [cited 2019 Sep 13]. https://www.tycho.pitt.edu/dataset/PH.38362002

2. Van Panhuis W, Cross A, Burke D, Choisy M. Counts of Dengue reported in Thailand: 1958–2010: Project Tycho dataset. University of Pittsburgh; 2018 [cited 2019 Sep 13]. https://www.tycho.pitt.edu/dataset/TH.38362002

3. Van Panhuis W, Cross A, Burke D, Choisy M. Counts of Dengue reported in Lao People's Democratic Republic 1979–2010: Project Tycho dataset [Internet]. University of Pittsburgh; 2018 [cited 2019 Sep 13]. https://www.tycho.pitt.edu/dataset/LA.38362002

4. Van Panhuis W, Cross A, Burke D, Choisy M. Counts of Dengue reported in Vietnam: 1960–2010: Project Tycho dataset [Internet]. University of Pittsburgh; 2018 [cited 2019 Sep 13]. https://www.tycho.pitt.edu/dataset/VN.38362002

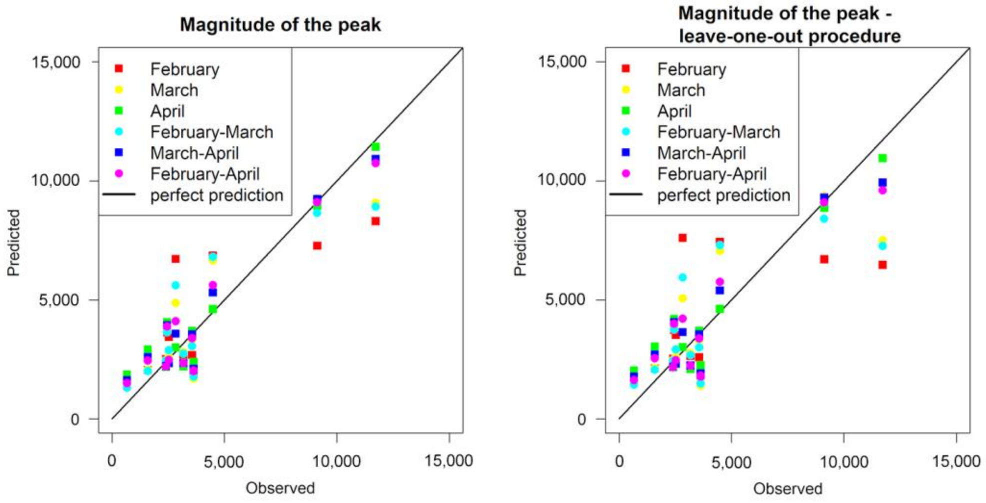

Appendix Figure 1. Observed versus predicted magnitude of the peak for each season using a simple linear regression model $M = \alpha + \beta N$, with M the magnitude of the peak and N the number of probable dengue cases reported to NDSS in April. The black line represents the expected results with perfect prediction. The figure on the right represents the results for the "leave-one-out" procedure.

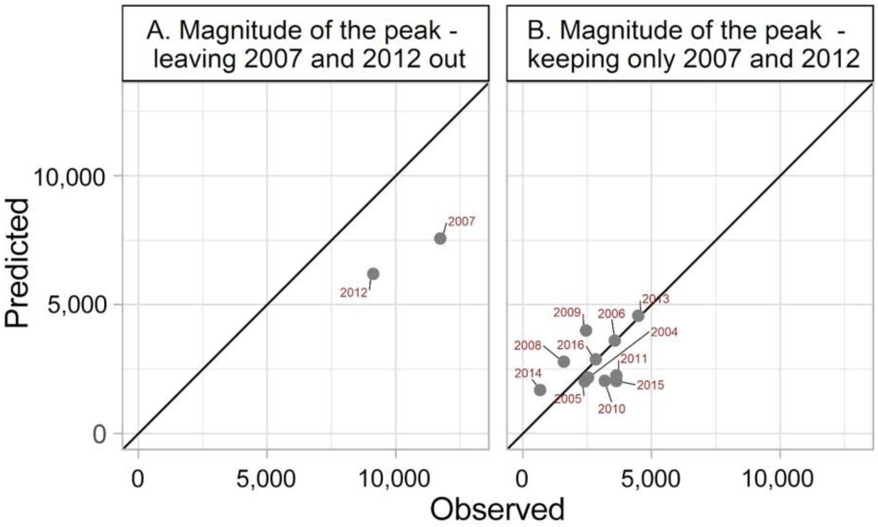

Appendix Figure 2. Observed versus predicted magnitude of the peak for each season using a simple linear regression model $M = \alpha + \beta N$, with M the magnitude of the peak and N the number of probable dengue cases reported to NDSS in April. The black line represents the expected results with perfect prediction. The figure on the left represents the results when we left 2007 and 2012 out of the learning sample and tried to predict the magnitude of the peak for these 2 years. Conversely, the figure on the right represent the results when we kept only 2007 and 2012 in the learning sample to predict the magnitude of the peak for the 11 other seasons.

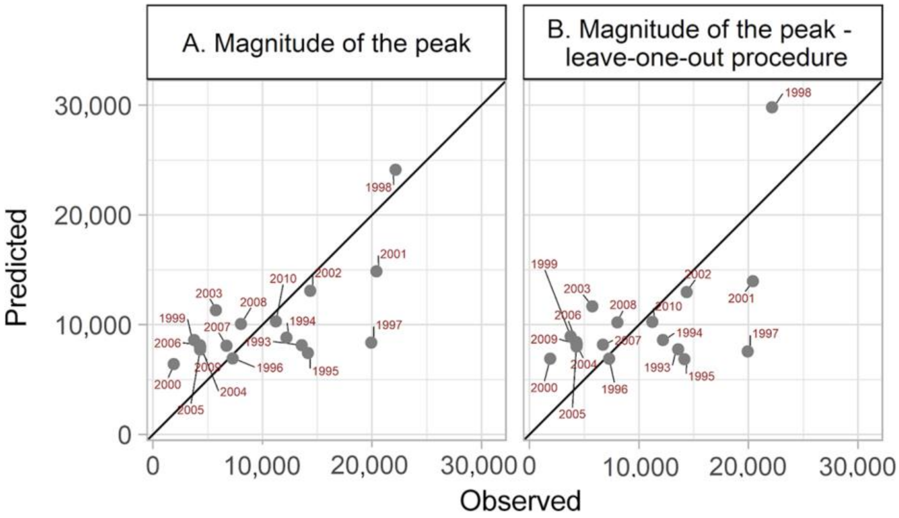

Appendix Figure 3. Observed *vs.* predicted magnitude of the peak for each season using a simple linear regression model $M = \alpha + \beta N$, with M the magnitude of the peak and N the number of reported dengue like cases in Thailand in February. The black line represents the expected results with perfect prediction. The figure on the right represents the results for the "leave-one-out" procedure.

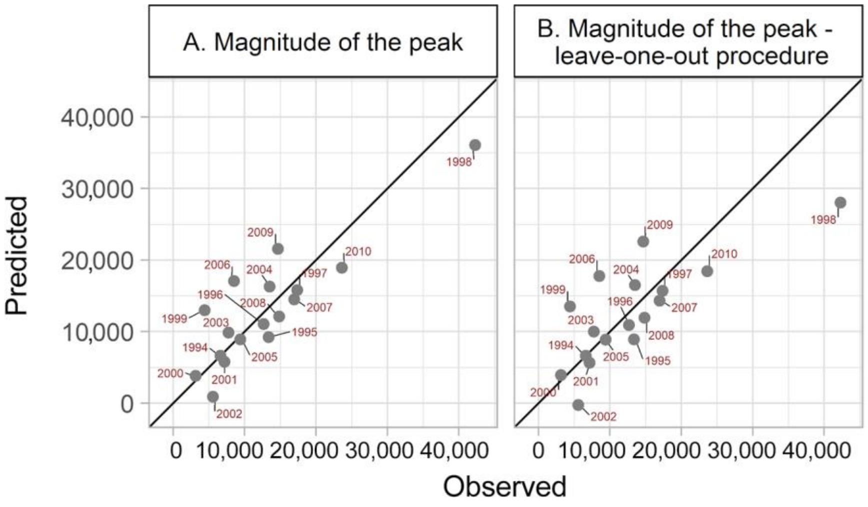

Appendix Figure 4. Observed *vs.* predicted magnitude of the peak for each season using a simple linear regression model $M = \alpha + \beta N$, with M the magnitude of the peak and N the number of reported dengue like cases in Vietnam in March. The black line represents the expected results with perfect prediction. The figure on the right represents the results for the "leave-one-out" procedure.

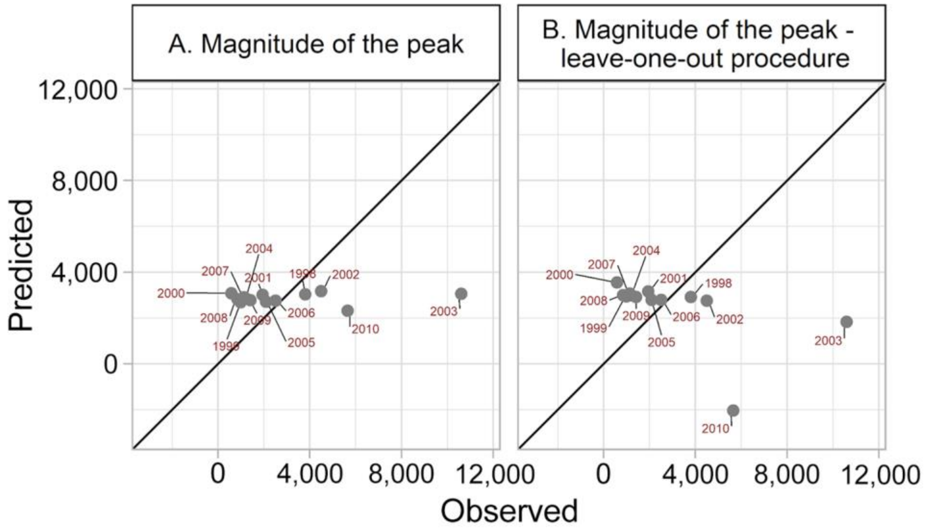

Appendix Figure 5. Observed *vs.* predicted magnitude of the peak for each season using a simple linear regression model $M = \alpha + \beta N$, with M the magnitude of the peak and N the number of reported dengue like cases in Laos in March. The black line represents the expected results with perfect prediction. The figure on the right represents the results for the "leave-one-out" procedure.

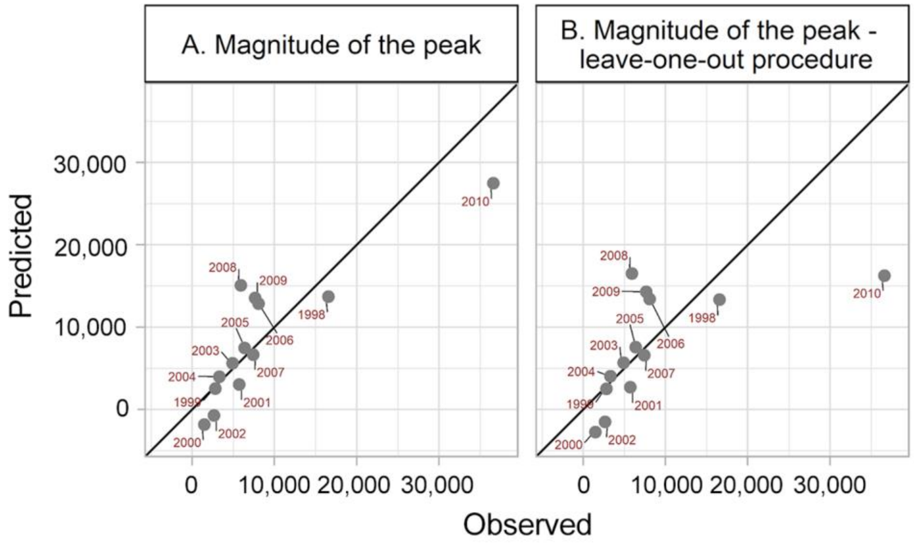

Appendix Figure 6. Observed *vs.* predicted magnitude of the peak for each season using a simple linear regression model $M = \alpha + \beta N$, with M the magnitude of the peak and N the number of reported dengue like cases in Philippines in May. The black line represents the expected results with perfect prediction. The figure on the right represents the results for the “leave-one-out” procedure.
